# Disseminated *Ureaplasma* infection: a case report of a patient with rheumatoid arthritis on rituximab therapy with multiple abscesses and arthritis

**DOI:** 10.48101/ujms.v131.13765

**Published:** 2026-03-19

**Authors:** Leonard Sindelar, Anna Hall, Göran Eriksson, David Lennebratt, Karlis Pauksens

**Affiliations:** a Department of Infectious Diseases, Gävle Hospital, Gävle, Sweden; b Department of Infectious Diseases, Västmanland County Hospital, Västerås, Sweden; c Department of Infectious Diseases, Falu Hospital, Falun, Sweden; d Department of Medical Sciences, Section of Infectious Diseases, Uppsala University Hospital, Uppsala, Sweden

**Keywords:** Disseminated *Ureaplasma*, immunosuppression, rheumatoid arthritis, rituximab, infection risk, anti-CD20

## Abstract

A 51-year-old woman with rheumatoid arthritis treated with rituximab and leflunomide presented with a 5-month history of malodorous vaginal discharge. Initial examination revealed vulvovaginitis and cervicitis, and she was treated with metronidazole and topical corticosteroids. Pelvic ultrasound showed free fluid in the pouch of Douglas, raising suspicion of ovarian malignancy. Shortly thereafter, she was hospitalized with fever, abdominal pain, and elevated C-reactive protein. Despite broad-spectrum intravenous antibiotics and multiple surgical drainages for recurrent intra-abdominal abscesses, the infection persisted, and repeated cultures were negative. Corticosteroids led to transient improvement, but new abscesses developed despite immunosuppressive therapy, and an aseptic abscess syndrome was suspected. Given her immunosuppressed status, further testing was undertaken, and polymerase chain reaction (PCR) analyses from urine, vaginal secretions, synovial fluid, and paraspinal abscess aspirate were positive for *Ureaplasma parvum*, for which her partner also tested positive.

Treatment with oral doxycycline led to rapid clinical and laboratory test improvement, and moxifloxacin was added for bactericidal coverage. Because of mild hypogammaglobulinemia, monthly intravenous immunoglobulin therapy was initiated.

This case illustrates the diagnostic challenges of *Ureaplasma* infections in immunocompromised patients, particularly those receiving B-cell-depleting therapy such as rituximab. Standard cultures fail to detect the organism, often delaying diagnosis. *Ureaplasma parvum* should therefore be considered in patients presenting with sterile abscesses, systemic inflammation, and urogenital symptoms. Early recognition through molecular testing and targeted antimicrobial treatment can prevent prolonged morbidity and unnecessary surgical interventions.

## Introduction

*Ureaplasma parvum* and *Ureaplasma urealyticum* are sexually transmitted bacteria capable of causing severe invasive infections in immunocompromised individuals. These organisms belong to the class *Mollicutes*, which lack a cell wall and are therefore undetectable using standard culture media. Detection relies on nucleic acid amplification tests, which can differentiate the two species known to infect humans: *Ureaplasma urealyticum* and *Ureaplasma parvum* (1). These bacteria are commonly found in the urogenital tract and may penetrate the submucosa in the presence of immunosuppression or mechanical injury. An international study reported colonization rates of up to 80% among healthy women (2), while a Swedish study from 2021 found that 33% of patients attending sexually transmitted infection (STI) clinic carried *Ureaplasma* species (3).

In cases of urethritis, *Ureaplasma parvum* is generally not considered a significant finding compared to *Ureaplasma urealyticum*. However, both species may have pathogenic potential in immunocompromised hosts (4). The most common manifestation of *Ureaplasma* infection in such individuals is septic arthritis. Three Swedish case reports of *Ureaplasma* infection included patients with prosthetic joint loosening, septic osteomyelitis with polyarthritis, and a patient on rituximab therapy who developed abdominal abscesses due to *Ureaplasma urealyticum* (5–7).

Our case report presents a disseminated *Ureaplasma parvum* infection with abscess formation in an immunocompromised patient. It underscores the diagnostic challenges associated with *Ureaplasma* species, particularly in the context of negative culture results and urogenital symptoms.

## Case report

A 51-year-old woman presented to the gynecology outpatient clinic at a county hospital in October 2024 due to foul-smelling vaginal discharge persisting for 5 months. The patient had a new sexual partner since February. On examination, she was found to have pronounced vulvitis/vaginitis and cervicitis. Treatment with metronidazole in combination with topical corticosteroids was initiated, and a follow-up transvaginal ultrasound was scheduled.

The patient had a prior history of anti-citrullinated protein antibodies (ACPA) and rheumatoid factor (RF)-positive rheumatoid arthritis, diagnosed in 2018, initially presenting with extensive pulmonary infiltrates considered an extra-articular manifestation of the disease. Initial treatment with etanercept and adalimumab was discontinued due to cutaneous adverse reactions. She was subsequently treated with leflunomide from 2018 and baricitinib from 2019. However, due to treatment failure, baricitinib was discontinued and replaced with rituximab on August 2021, administered at a dose of 500 mg every 6 months. The patient subsequently achieved remission on a regimen of rituximab and leflunomide. Prior to the illness in autumn 2024, the patient was completely asymptomatic in her joints, except for thumb discomfort in late autumn, which resolved after a short course of prednisolone.

During the subsequent ultrasound examination, fluid was detected in the pouch of Douglas, prompting initiation of a standardized care pathway (SVF) for suspected ovarian cancer. Three days later, the patient was admitted acutely with fever, abdominal pain, and elevated C-reactive protein (CRP) of 161 mg/L and a leukocyte count of 14 × 10⁹/L, and treatment with intravenous piperacillin/tazobactam was initiated. In previous rheumatoid arthritis flares, CRP and leukocyte counts were typically not elevated, and the most recent dose of rituximab had been administered 2 months earlier.

Due to continued elevation of CRP, aspiration of a suspected abscess and fluid from the pouch of Douglas was performed. As fluid reaccumulated in the pouch, a drain was placed, and samples were sent for routine culture as well as for tuberculosis, *Actinomyces*, and *Nocardia*.

One day after the drain placement, *Enterococcus raffinosus* was cultured from the drainage fluid, resistant to piperacillin/tazobactam. Antibiotic therapy was switched to imipenem combined with vancomycin. The patient showed clinical improvement and inflammatory markers decreased. Vancomycin was discontinued after 1 week, and imipenem monotherapy was continued. Follow-up abdominal computerized tomography (CT) revealed a new fluid collection adjacent to the sigmoid colon, suggestive of an abscess. Due to this therapeutic failure, laparoscopic drainage was performed. Postoperatively, the patient deteriorated with rising CRP and fever, prompting exploratory laparotomy. Intraoperatively, serosal tears near the sigmoid colon and small intestine were identified, leading to bilateral salpingo-oophorectomy. The appendix could not be visualized because of suspected necrosis, and a loop ileostomy was applied to optimize healing. Two passive drains were placed in the abdomen.

Persistent postoperative fever led to broadening of the antimicrobial coverage with caspofungin and reinitiation of vancomycin. Despite this, inflammation persisted, and betamethasone (Betapred) was initiated. Within 3 days, CRP decreased from >300 mg/L to 36 mg/L, and the patient became afebrile.

At a subsequent multidisciplinary conference, antifungal therapy and imipenem were discontinued, while vancomycin was continued due to clinical and laboratory response. Histopathology from intraoperative biopsies revealed neutrophilic infiltrates and necrosis consistent with abscess formation. During the hospital stay, she experienced gastrointestinal symptoms, but no joint complaints or skin rashes were noted. Inflammatory bowel disease (IBD) was considered as a differential diagnosis. After 2 weeks, vancomycin was discontinued as the patient remained afebrile and CRP continued to decline. However, wound dehiscence was noted at the midline laparotomy site, and *Klebsiella pneumoniae* was cultured from the wound. Intravenous treatment with cefotaxime was initiated. CT performed 3 days later showed progressive abscess formation, prompting a switch from cefotaxime to vancomycin.

At a later conference, aseptic abscess syndrome was considered due to the development of new abscesses despite broad-spectrum antibiotic therapy and repeated negative cultures. Once again, the patient responded rapidly to betamethasone treatment with declining CRP and resolution of fever. After reviewing the literature (several case reports) and discussing the case with colleagues at the multidisciplinary conference (Gynecology, Gastroenterology, and Infectious Diseases), it was decided to trial tumor necrosis factor (TNF) inhibitor therapy. Infliximab was chosen due to prior experience with the drug in similar cases of aseptic abscess syndrome, as well as previous cutaneous reactions to adalimumab and etanercept. This decision, along with prior reactions to TNF inhibitors, was discussed with dermatology colleagues, who deemed it acceptable to trial infliximab. Treatment with vancomycin was continued due to residual concern for infectious etiology. After 3 months of hospitalization, the patient was discharged with outpatient treatment using dalbavancin.

In early January 2025, Uppsala University Hospital was contacted due to continued uncertainty regarding the etiology of the abscesses. The infectious disease consultant recommended to consider testing for *Ureaplasma*, *Burkholderia cepacia*, and investigate for chronic granulomatous disease. Workup for chronic granulomatous disease was normal, and further testing was deferred due to clinical improvement and low CRP.

In late January, the patient developed arthritis near the right thumb and bilateral trochanteritis. CRP increased again, and an abdominal CT showed a slight accumulation of fluid in the pouch of Douglas. Treatment with methotrexate was initiated on February 6th 2025. At that time, she was receiving a combination of infliximab and leflunomide.

Due to a lack of improvement, the patient was readmitted with CRP 146 mg/L and newly identified arthritis in both wrists and metacarpophalangeal joints (MCP) I bilaterally. A phlegmon in the left gluteal region was also identified. Dalbavancin was continued, with a partial response in inflammatory markers. In late February, the patient was transferred to the rheumatology department in Uppsala for further evaluation of suspected aseptic abscess syndrome, as the condition had progressed despite immunosuppressive therapy and local steroid injections.

A CT of the abdomen and pelvis on February 28 revealed fluid accumulation near the right L3/L4 facet joint, bilateral trochanteric bursitis, and edema/phlegmon in the left gluteal region. Given the initial vaginal symptoms and immunosuppressed status, *Ureaplasma* infection was suspected again. *Whipple’s disease*, *Bartonella*, and *Burkholderia cepacia* were also considered. Samples were then collected from urine, vaginal secretions, synovial fluid, and the L3/L4 fluid collection for PCR testing. Methotrexate, as well as infliximab and leflunomide, was paused, and oral doxycycline therapy was initiated on March 2.

After 3–4 days of treatment, the patient showed clinical improvement, and CRP declined. PCR analysis was positive for *Ureaplasma parvum* in urine, vaginal secretions, synovial fluid from the wrist, and the spinal fluid collection. The patient’s partner also tested positive in urine, and eradication therapy with doxycycline was recommended to prevent reinfection. At discharge, the patient had reduced pain, improved mobility, and enhanced ability to perform activities of daily living (ADLs). Residual hand pain persisted but showed gradual improvement. No new joint involvement was observed. The patient was able to partially resume work from home.

Moxifloxacin was added to doxycycline to achieve a bactericidal effect with a fluoroquinolone. The preliminary treatment duration was planned for 3 months. Given the patient’s mild hypogammaglobulinemia (IgG 5.8 g/L), intravenous immunoglobulin (IVIG) was administered at a dose of 0.5 g/kg from 31st of March 2025 for every 4 weeks during the course of infection treatment, based on evidence linking hypogammaglobulinemia to increased severity of *Ureaplasma* infections. The patient’s IgG levels had remained above 12 g/L for the previous 10 years until 2022, after which they declined, likely as a result of rituximab therapy.

The initial plan was to continue antibiotic treatment for at least 3 months; however, due to limited guidelines and the risk of recurrence, therapy was extended to 6 months. At follow-up, the patient was well, without signs of infection. CRP levels were repeatedly within the normal range after cessation of antibiotics. The patient experienced occasional arthritis thereafter, which was considered related to underlying rheumatoid arthritis. Local corticosteroid treatment was effective, and disease-modifying antirheumatic drug therapy was not reintroduced at that time although plans for reinitiation of methotrexate are being considered. The patient has continued to receive immunoglobulin therapy, with ongoing evaluation of treatment duration based on infection frequency and immunoglobulin levels.

## Discussion

Treatment of *Ureaplasma* infection is initially often empirical, as the organism is primarily detected via molecular methods. Susceptibility to tetracyclines, macrolides, and fluoroquinolones has generally been assumed in Europe although global resistance is increasing (8). A French study reported 8% tetracycline resistance among clinical isolates (9). No international treatment guidelines currently exist. Treatment recommendations for *Ureaplasma* infections are largely based on in vitro susceptibility data. In extragenital infections, fluoroquinolones such as moxifloxacin and levofloxacin are preferred as first-line agents due to their bactericidal properties (10). Doxycycline is considered a suitable alternative, particularly in cases where fluoroquinolones are contraindicated.

Although there is no definitive evidence supporting combination therapy, several case reports suggest improved clinical outcomes compared to monotherapy, especially in immunocompromised patients (11). Combination therapy may potentially reduce the risk of resistance development.

Hypogammaglobulinemia appears to be a risk factor for opportunistic invasive infections caused by *Ureaplasma*, as supported by multiple case reports involving patients with conditions such as common variable immunodeficiency (CVID) or secondary hypogammaglobulinemia following anti-CD20 antibody therapy. In patients with hypogammaglobulinemia, immunoglobulin replacement therapy should be considered as a preventive or therapeutic measure.

A common diagnostic pitfall in *Ureaplasma* infections among patients treated with rituximab is the inability to culture the organism using standard methods, often resulting in delayed diagnosis. Several case reports emphasize that patients had a history of urinary tract symptoms prior to developing disseminated infection (12).

## Conclusion

This case illustrates a clinical course beginning with urogenital symptoms following sexual contact with a new partner, progressing to disseminated infection with abscess formation in an immunocompromised patient. Positive PCR findings for *Ureaplasma parvum* from multiple sites – including urine, genital samples, synovial fluid, and abscess material – collectively supported the diagnosis of *Ureaplasma parvum* infection. The case highlights the importance of including *Ureaplasma* in the differential diagnosis of systemic infections in immunocompromised individuals, particularly in the presence of urogenital symptoms and negative culture results.

## Registration and consent

Consent for publication was obtained from the participant.
